# The ultrasound-guided funicular block in cats undergoing orchiectomy: ropivacaine injection into the spermatic cord to improve intra and postoperative analgesia

**DOI:** 10.1186/s12917-022-03279-4

**Published:** 2022-05-10

**Authors:** Vincenzo Cicirelli, Burgio Matteo, Caterina Di Bella, Giovanni Michele Lacalandra, Giulio Aiudi

**Affiliations:** 1 Department of Veterinary Medicine, University of Bari “Aldo Moro”, Bari, Italy; 2 School of Biosciences and Veterinary Medicine, University of Camerino, Matelica, Italy

**Keywords:** UGF block, Orchiectomy, Ropivacaine, Local analgesia

## Abstract

**Background:**

The orchiectomy in cats is a common surgical procedure with medium level of pain and for this reason requires intra and postoperative analgesia management. The aim of this study was to compare intra and postoperative pain in two groups of cats undergoing orchiectomy. Sixty healthy cats were randomly assigned in two groups (*n* = 30) to receive pre surgery ropivacaine hydrochloride (0.2 mL/kg at 0.5%) (R Group) or NaCl 0.9% (C group) into the spermatic cord. The intraoperative evaluation was carried out using the cardiorespiratory stability parameters and eventually administration of rescue analgesia. A rescue analgesia (fentanyl 2 µg/kg) was administered during orchiectomy in case of considerable increase of blood pressure, heart rate or respiratory rate. The postoperative evaluation was been done using scores following a UNESP-Botucatu multimodal scale for 6 h post-surgery.

**Results:**

As result, cats in R group responded better to surgical procedure, maintaining lower postoperative pain scores than C group.

**Conclusions:**

The ultrasound-guided funicular block used in this study, as already demonstrated in dogs, is a good method to protect the cats from surgical pain and ensure a good level of surgical analgesia.

## Introduction

Cat orchiectomy is a surgical procedure that requires an effective perioperative analgesia [1]. Surgical analgesia is very important because pain can delay recovery, decrease quality of life, disturb the human–animal bond, increases the body’s stress response to traumatic injury and causes alterations in metabolic and endocrine function [2, 3]. Several authors have already described loco-regional anaesthetic as testicular block, the injection of local anaesthetics into the testicular parenchyma, to ensure a good pain protection during surgical neutering in domestic animals [4, 5]. In fact, local anaesthetics provide effective surgical analgesia, blocking the transmission of pain without side effects [6]. The ropivacaine, tested for various anaesthetic block [7], was used in ultrasound-guided funicular (UGF) block in dog surgical neutering. The UGF block is the infiltration of ropivacaine into the spermatic cord to optimize the action of the local anaesthetic [8]. This study stems from the idea that this block, as already described in the dog, added to general anaesthesia may help control pain, and reduce the need for rescue analgesia during surgery and in postoperative period [8]. This study aimed to evaluate the analgesic efficacy of UGF block in cats, considering: intraoperative pain (intended as cardiorespiratory stability and administration of additional analgesic drugs), and postoperative pain scores following the UNESP-Botucatu multimodal scale. This scale, developed using a psychometric methodology, takes into account postural attitudes and facial expressions, vocalizations and mood, the response to manipulation and palpation of the painful area, physical activity and gait, and the subject's behavior towards the surrounding environment [9–11]. This scale was considered the most appropriate for the type of evaluation performed in this study. We aimed to compare the UGF block and routine general anaesthesia using intraoperative cardiorespiratory stability and postoperative pain scores. Therefore, we hypothesised that the UGF block improves analgesia during cat’s orchiectomy as already demonstrated in the dog.

## Materials and methods

### Study design

This was an assessor-blinded, randomized, clinical research study. All cases were enrolled over a 6 week period during the autumn of 2021 at Clinic of the Veterinary Hospital of the ‘Aldo Moro’ University of Bari. The same team of surgeon performed all the procedures.

### Animals

Sixty male cats presented for castration, weighing 2, 5 – 4, 1 kg with a age < 1 years, were recruited to this study after obtaining informed owner consent and approval from the ‘Aldo Moro’ University of Bari ethical committee (Approval Number 15/2021). They were of good health, had no previous pathologies, and were allocated to the very low aesthetic risk class (ASA 1). Exclusion criteria were cats that had been treated with any analgesic, sedative or anesthetic drug in the previous 30 days, very agitated / aggressive, obese and with clinical signs of disease. Two days before surgery, patients underwent a comprehensive physical examination, hematological and serum biochemistry panel. The cats were randomly assigned to two groups: the R and C groups, using StatView statistical software.

### Pre-surgery procedure

In both groups, the cats were sedated using intramuscular injections of 3 mcg/kg dexmedetomidine (Dexdomitor®, Vetoquinol Italia SRL, Bertinoro, Italy) and 0.25 mg/kg methadone (Semfortan®, Eurovet Animal Health BV, Bladel, The Netherlands) mixed in the same syringe [12]. The premedicants were administered into the lumbar epaxial muscles. After 20 min, a 24-G venous catheter was inserted to start a standard maintenance fluid therapy (NaCl 0.9%, 4 ml/Kg/h) [13]. Propofol (Vetofol®, Esteve, Barcelona, Spain) at 1 mg/kg was administered intravenously to induce general anesthesia. Orotracheal intubation was promoted, while anaesthetic maintenance was performed with sevoflurane (EtSev 2,5%, SevoFlo®, Ecuphar Italia S.r.l., Milano, Italy) [14], vaporized in 100% oxygen, in an open anaesthesia system, always performed by the same anaesthesiologists. From this point throughout the surgery, an anesthetist blinded to the group monitored: plane of anesthesia, spontaneous ventilation, heart rate, respiratory rate, non-invasive blood pressure, oxygen hemoglobin saturation and body temperature (monitor GE-Datex Ohmeda B 450), ensuring a satisfactory anesthetic plan [15, 16].

### UGF block (R group) and NaCl 0.9% injection (C group)

The UGF block was performed with the guidance of an ultrasound system (MyLab™ ClassC, Esaote Spa, Genua, Italy). The infiltration of ropivacaina (0.2 mL/kg at 0.5%) (Naropina®, Aspen Pharma Trading 69 Limited) was performed using a BD needle with a Quincke tip (22 G, 0.7 × 90 mm). The local anesthetic was infiltrated into the spermatic cord at the level of its emergence from the superficial inguinal ring (R group) [8]. In C group the same volume of NaCl 0.9% was injected in the same way.

### Surgery procedure

The same surgical team orchiectomized all 60 cats. All surgery were performed with a scrotal approach and lasted about 6 min (± 30 s). Before the procedure, the cardiorespiratory parameters of all animals (pre-incisional values of heart rate, respiratory rate, and blood pressure) were recorded to evaluation the eventual surgical increase [17]. These parameters were registered at six moments during the procedure: first skin cutting of the scrotum (S1), traction on the first spermatic cord (S2), ligature of the spermatic cord (S3), second skin cutting of the scrotum (S4), traction of the second spermatic cord (S5) and ligature of the second spermatid cord (S6). In case of intraoperative increase of 30% of cardiorespiratory parameters respect the pre-incisional value, a bolus of fentanyl was administered i.v. (2 mcg/kg, Fentadon®, Eurovet Animal Health BV) [18]. At the end of the surgery, 0.2 mg of Meloxicam® (Metacam, Boehringer Ingelheim Italia S.p.A.) was injected s.c. in all patients [19].

### Postoperative pain evaluation

Postoperative evaluations began at the end of surgery (0 h) and were repeated at 1, 2, 3, 4, 5 and 6 h. In this period, a blind operator to the group the cat belonged, assigned a score from 0 (pain free) to 30 (unbearable pain), using the UNESP-Botucatu scale [10, 11]. Patients with 7 or higher scores received methadone hydrochloride (0.2 mg/kg im) as rescue analgesia [10, 11].

### Data analysis

Data analysis was performed using Stata MP17 software and compiled forms were imputed into a dataset using an Excel spreadsheet. Continuous variables were described as mean ± standard deviation (SD) and range, and categorical variables as proportions. The skewness and kurtosis test was utilized to evaluate the normality of continuous variables; all the continuous variables were normally distributed. The t student test for independent data was used to confront continuous variables between groups, the ANOVA for repeated measures test was used to compare continuous variables between groups and detection time; the Fisher’s exact test were used to compare the proportions. To assess the determinants of rescue analgesia, a multivariate logistic regression model was used in which rescue analgesia was the outcome and group assignment (R vs. C), age (months) and weight (kg) were the determinants. The adjusted odds ratio (aOR) was calculated with the 95% confidence interval (95%CI). For all tests, a p-value < 0.05 was considered statistically significant.

## Results

Table 1 describes the characteristics of the sample, by group. The study sample consisted of 60 cats: 30 (50.0%) in the C group and 30 (50.0%) in the R group.

**Table 1:** Sample characteristics by group (C vs. R)

Variable	C (*n* = 30)	R (*n* = 30)	Tot. (*n* = 60)	*p*-value
Age (years)	8.4 ± 1.8	8.7 ± 2.1	8.6 ± 1.9	0.653
	(6–12)	(6–12)	(6–12)	
Weight (kg)	3.2 ± 0.5	3.3 ± 0.5	3.2 ± 0.5	0.473
	(2.5–4.1)	(2.5–4.1)	(2.5–4.1)	

**Fig. 1:**
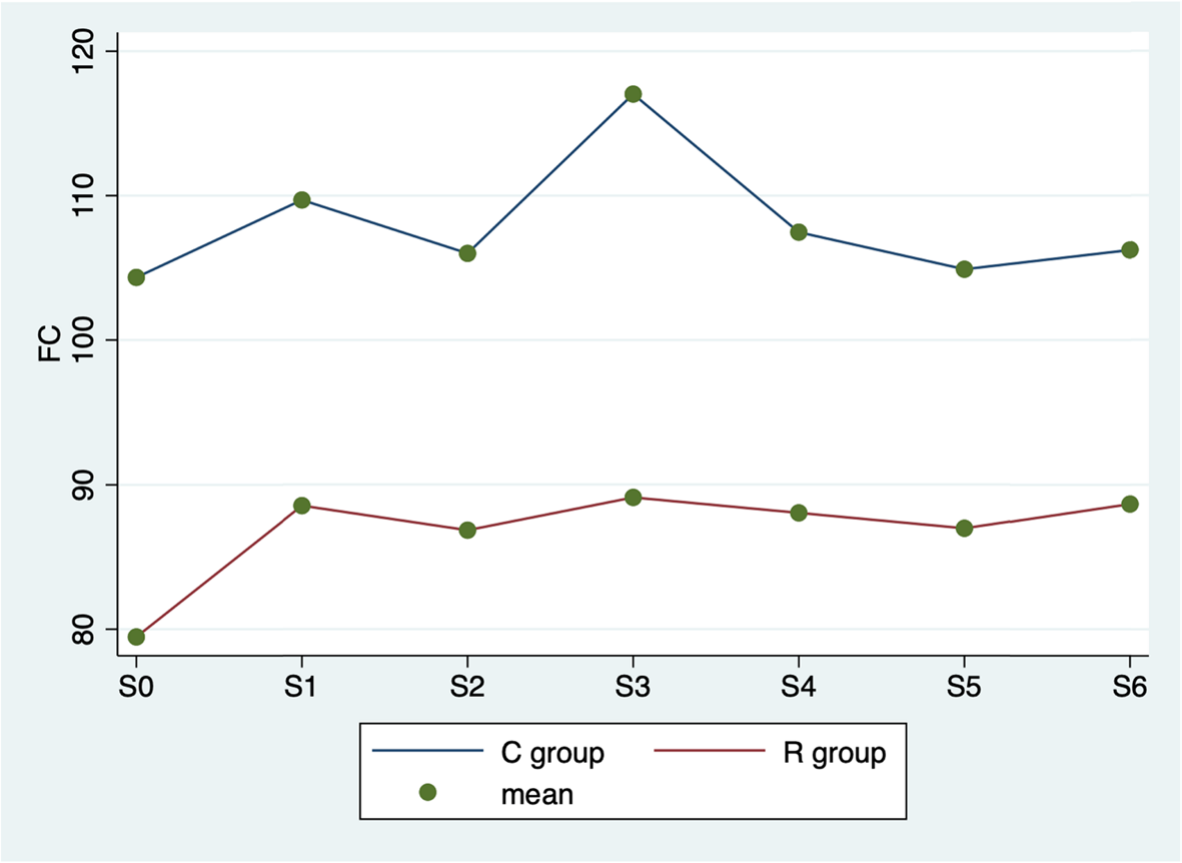
ANOVA test. Average heart rate values by group (C vs. R) and detection time

In Fig. 1 ANOVA test highlights significant differences in the comparison of heart rate among different times (*p* < 0.0001), groups (*p* < 0.0001) and interaction between time and group (*p* = 0.025).

**Fig. 2:**
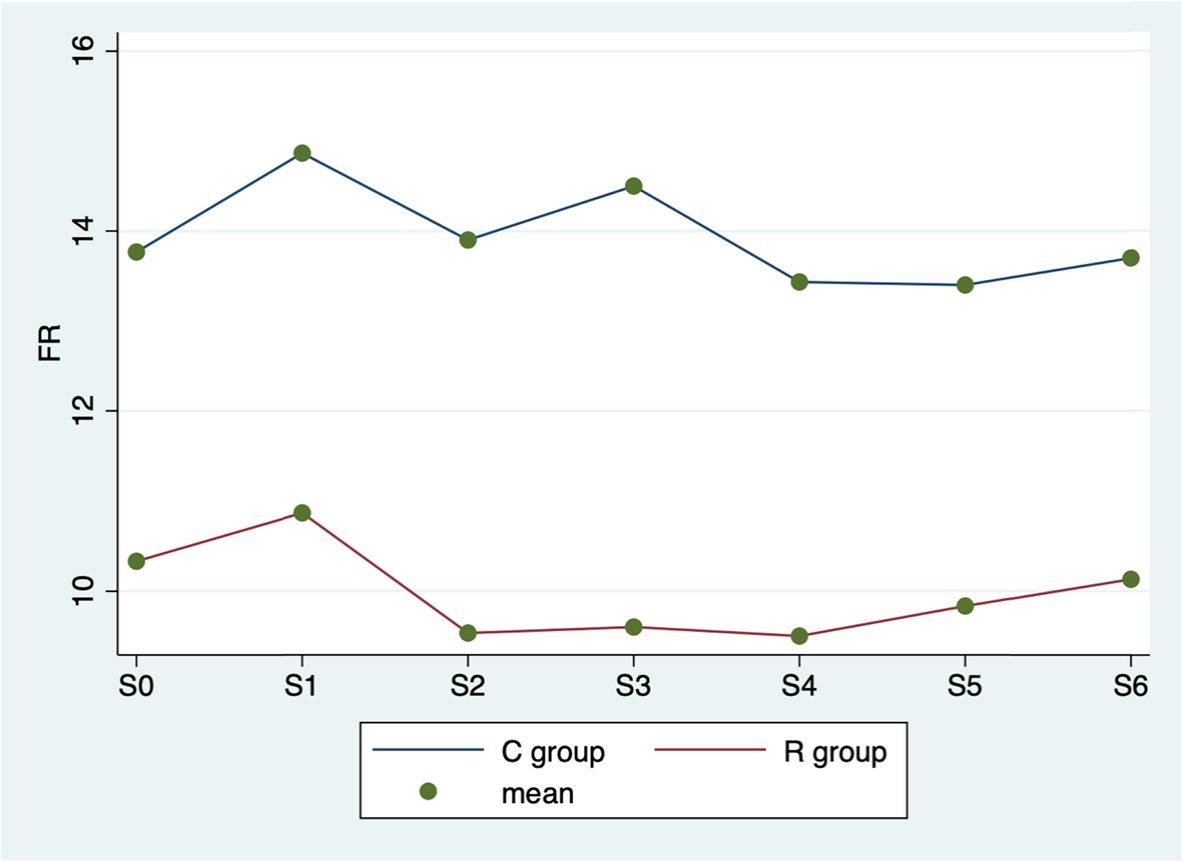
ANOVA test. Average respiratory rate by group (C vs. R) and detection time

In Fig. 2 ANOVA test showed significant differences in respiratory rate among various times (*p* = 0.001), between groups (*p* < 0.0001), but not in the interaction between time and group (*p* = 0.267).

**Fig. 3:**
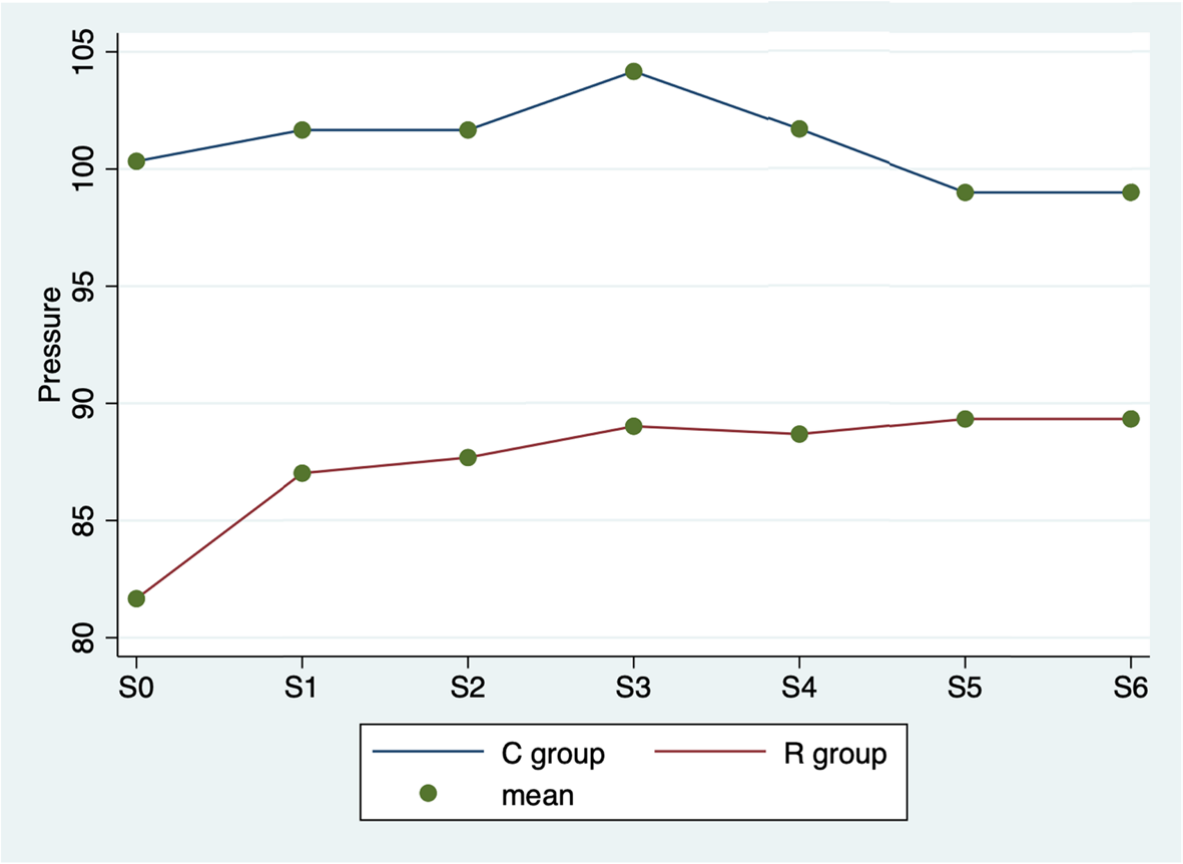
ANOVA test. Average blood arterial non invasive pressure values by group (C vs. R) and detection time

In Fig. 3 ANOVA test showed a significant difference in arterial blood non invasive pressure values among the various times (*p* < 0.0001), between groups (*p* < 0.0001) and in the interaction between time and group (*p* < 0.0001).

**Fig. 4:**
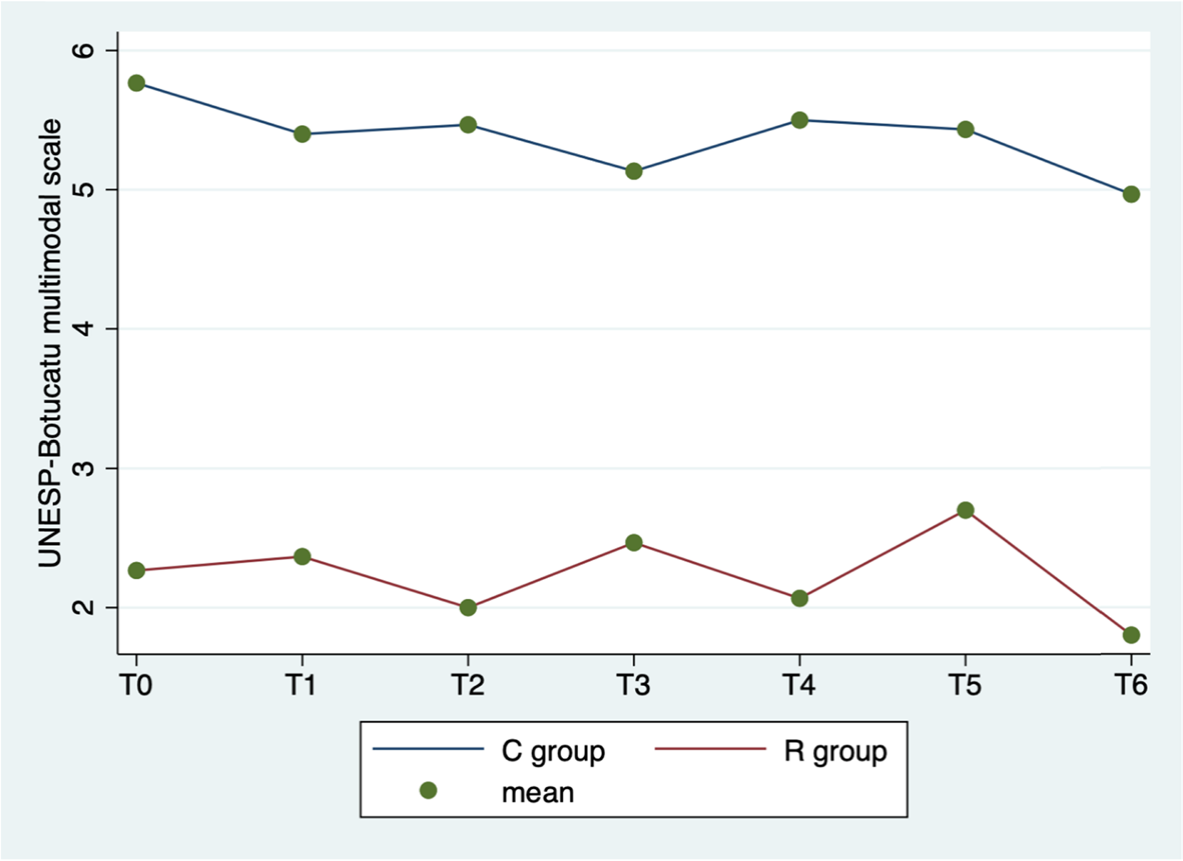
Average UNESP-Botucatu scores by group (C vs. R) at different detection times

In Fig. 4 ANOVA test showed significant differences between groups in the UNESP-Botucatu scores (*p* < 0.0001), detection times (*p* < 0.0004), but not in the interaction between time and group (*p* = 0.071).

Table 2. describes the observed events related to intraoperative fentanyl administration.

**Table 2:** Proportion of cats undergoing surgical fentanyl administration, by group and time of detection

Variable	C (*n* = 30)	R (*n* = 30)	Tot. (*n* = 60)	*p*-value
S1	6 (20.0%)	3 (10.0%)	9 (15.0%)	0.472
S3	6 (20.0%)	1 (3.3%)	7 (11.7%)	0.103
S4	1 (3.3%)	0 (0.0%)	1 (1.7%)	1.000
S6	1 (3.3%)	0 (0.0%)	1 (1.7%)	1.000
Tot	14 (46,7)	4 (13,3)	18 (30,0)	0,005

In Table 3 a multivariate analysis highlighted a statistically significant association between postoperative methadone administration and group (aOR = 0.18; 95%CI = 0.05–0.64); no further associations were observed (*p* > 0.05).

**Table 3:** Analysis of the determinants of rescue analgesia administration (methadone) in a multivariate logistic regression model

Determinant	aOR	95%CI	*p*-value
Group (R vs. C)	0.18	0.05–0.64	0.008
Age (years)	1.04	0.74–1.45	0.829
Weight (kg)	0.84	0.23–3.08	0.791

The proportion of cats undergoing postoperative rescue analgesia (methadone) by group and time of detection is described in Table 4.

**Table 4:** Rescue analgesia (methadone) administration by group and detection time

Variable	C (*n* = 30)	R (*n* = 30)	Tot. (*n* = 60)	*p*-value
T0	3 (10.0%)	0 (0.0%)	3 (5.0%)	0.237
T2	1 (3.3%)	0 (0.0%)	1 (1.7%)	1.000
T3	2 (6.7%)	2 (6.7%)	4 (6.7%)	1.000
T6	3 (10.0%)	0 (0.0%)	3 (5.0%)	0.237
Tot	9 (30.0)	2 (6.7)	11 (18.3)	0.020

In Table 5 a multivariate analysis indicated a statistically significant association between postoperative methadone administration and group (aOR = 0.18; 95%CI = 0.03–0.92); no further associations were observed between outcomes and determinants (*p* > 0.05).

**Table 5:** Analysis of the determinants of postoperative rescue analgesia administration in a multivariate logistic regression model

Determinant	aOR	95%CI	*p*-value
Group (R vs. C)	0.18	0.03–0.92	0.039
Age (years)	0.84	0.56–1.25	0.387
Weight (kg)	0.52	0.11–2.47	0.411

## Discussion

Surgical analgesia and its management continue to be popular topics for discussion in the field of veterinarian anaesthesiology [20]. It is important to ensure that the safest and most efficacious methods are being utilized to treat pain in the perioperative period. Inadequately controlled surgical pain can have a significant impact on patients’ recovery and quality of life [21]. In this study, the use of UGF block during cat orchiectomy was investigated to evaluate his analgesic efficacy, considering intraoperative pain and postoperative pain scores following a UNESP-Botucatu multimodal scale. The results of this study show that, compared to the pre-incisional values, during the procedure the parameters considered often tend to increase in both groups. Normally cat orchiectomy induced significant haemodynamic responses, intended as heart rate, respiratory rate, and blood pressure variation. The correlation between these parameters used in this study and intraoperative pain is commonly used in veterinary medicine [22]. In fact, when a patient receives a surgical pain, sympathetic nervous system stimulate the increase of the heart rate, respiratory rate, and blood pressure [23, 24]. The results show that in R group the values considered for the evaluation of the intraoperative analgesic plan are better. Figures 1, 2 and 3 show that the UGF block before the cat orchiectomy, reduce the intra e postoperative surgical pain. The results of this study highlights that the ropivacaine administered in the inguinal ring on the tissues close to the genital femoral nerve, is quickly distributed to the spermatic cord thus improving the analgesic plan of the cat orchiectomy, as already demonstrated in the dog [8]. In addition, this technique reduces considerably the use of intraoperative rescue analgesia (Table 2). The aim of the veterinary team participating in the procedure was on achieving a good analgesic plan in all study subjects. A fundamental part of modern medicine veterinary care is prevention and management of surgical pain. For this reason, anaesthetists have begun to apply improved protocols that aim to relieve surgical pain and thus improve surgical outcomes [25, 26]. These new techniques, used in this study for the R group ad called multimodal analgesia, include combinations of drugs with different dosages, routes of administration and timing. The American Society of Anesthesiologists recommends management of surgical pain using a multimodal approach [27] because drugs with differing mechanisms of actions target pain pathways resulting in additive and/or synergistic effects [28]. In addition, the use of UGF block also resulted in less UNESP-Botucatu scores in 6 h post-surgery. In fact, the use of ropivacaine provides good analgesic support for a long time in postoperative period [7], ensuring better quality of convalescence and reducing the need for another analgesic (Table 4). Ropivacaine has been widely used in humans in recent years, and is becoming increasingly popular in veterinary medicine [7]. This local anaesthetic led to no significantly aversive events in any animals of the studies, it is relatively inexpensive, and easy to use. In addition, it contributes to the postoperative well‐being of cat undergoing orchiectomy, and offers the advantages of being non-invasive while guaranteeing a sufficient analgesic effect for a sufficient period. In this research, all 60 cats were neutered under general anaesthesia without any surgery complications; this evidence the safety of UGF block. It is also notable that the duration of surgery did not vary in two groups; no surgeries exceeded 6 min. This underlines that the use of UGF block does not affect the duration of the cats orchiectomy. As is known, the pain assessment in cats is not easy. Several authors reported the difficulty in recognizing pain in the feline species [29]. In fact, neuroendocrine parameters, as norepinephrine, epinephrine, cortisol, and blood sugar levels, occur in response to sympathetic stimulation caused in part by pain. However, clinical experience should be used when assessing these objective measures of pain because fear, stress, anaesthesia, and pharmacologic interventions also cause these indicators to change [30]. In this study, we have chosen cat’s behavioural observations, rather than neuroendocrine tests, as indicators of postoperative pain [31]. The UNESP-Botucatu pain scale [9–11], already used in several studies in cats, was chosen in this experiment because is helpful and practical for the identification of pain in cats.

## Conclusions

The present study demonstrated that UGF block using ropivacaine confers satisfactory intra and postoperative analgesia during orchiectomy in cats. Considering the cost, availability, restrictions, and side effects of ropicavaine, routine use of this block is considered desirable in in daily clinical practice.

## Data Availability

All data generated or analyzed during this study are included in this article and are available from the corresponding author on reasonable request.
